# Complete genome sequence of *Mammaliicoccus lentus* (*Staphylococcus lentus*) strain B3, isolated from mouse early-life gut microbiota

**DOI:** 10.1128/mra.00214-25

**Published:** 2025-05-27

**Authors:** Dongqing Xu, Yining Liu, Songfei Guo, Fengyi Wan

**Affiliations:** 1 Department of Biochemistry and Molecular Biology, Bloomberg School of Public Health, Johns Hopkins University 25802, Baltimore, Maryland, USA; 2 Department of Molecular Microbiology and Immunology, Bloomberg School of Public Health, Johns Hopkins University 25802, Baltimore, Maryland, USA; 3 Sidney Kimmel Comprehensive Cancer Center, Johns Hopkins University 1466, Baltimore, Maryland, USA; University of Maryland School of Medicine, Baltimore, Maryland, USA

**Keywords:** *Staphylococcus lentus*, early life, gut microbiota, genome

## Abstract

We report the complete genome sequence of *Mammaliicoccus lentus* (*Staphylococcus lentus*) strain B3, a gram-positive bacterium isolated from mouse early-life gut microbiota that activates breast milk complement components. The circular genome consists of 2,813,148 base pairs, encompassing 3,711 predicted protein-coding sequences and 79 RNA genes.

## ANNOUNCEMENT

*Mammaliicoccus lentus* (*Staphylococcus lentus*), a coagulase-negative member of the *Staphylococcaceae* family, belongs to the *Mammaliicoccus sciuri* (*Staphylococcus sciuri*) group (1) and the novel genus *Mammaliicoccus,* closely related to *Staphylococcus* (2). While isolated from some animals (3) and proposed as the causative organism in clinical case reports (4–6), *M. lentus* is considered a commensal microbe in the skin (7) and gut (8) microbiota during early life.

We present the complete genome sequence of a gram-positive bacterium *M. lentus* (*S. lentus*) strain B3, isolated from the gut microbiota of 21-day-old complement component C1qc knockout (*C1qc*^-/-^) mice at Johns Hopkins University, Baltimore, MD, USA, according to the IACUC-approved protocol MO22H147. The bacterium was identified when cecal and colonic contents of *C1qc*^-/-^ mice were resuspended in sterile phosphate-buffered saline and plated on Luria–Bertani (LB) agar plates, followed by an aerobic incubation for 16 h at 37°C (8). To isolate single colonies of this bacterium, we employed repeated streak plating on LB agar plates.

Bacterial culture from a single colony in LB broth at 37°C with aeration for 16 h was used for genomic DNA isolation with a NucleoSpin Tissue Kit (Macherey-Nagel, Bethlehem, PA, USA). DNA quantity and quality were determined using a Qubit 4 Fluorometer (Thermo Fisher Scientific, Waltham, MA, USA).

Genome sequencing was performed at Novogene Corporation (Sacramento, CA, USA). Samples for single-molecule real-time (SMRT) sequencing were prepared with a PacBio Sequel II DNA CLR Library Kit (Pacific Biosciences, Menlo Park, CA, USA). The quantified libraries were sequenced on PacBio Sequel II/IIe systems, with continuous long-read mode.

The sequencing data were processed and filtered using the software SMRTLink (9) version 7.0, with minLength 0 and minReadScore 0.8 as parameters. Polymerase reads were trimmed to include only the high-quality region, and each polymerase read was partitioned to form one or more subreads that contain the full set of quality values and kinetic measurements. The SMRT long read sequencing (*N*_50_ = 17,678) resulted in 190,215 reads, with 1,037× coverage. The initial genome assembly was carried out using the software Canu (10) version 2.3 with the parameter genomeSize = 2.8 m, and circularity determination was performed using the software Circlator (11) version 1.5.5, with initiation from the start codon of the *dnaA* gene. The newly assembled genome was further improved with our recently published Illumina sequencing data, 30,126,990 reads quality controlled using the software Trimmomatic (12) version 0.36 (8), using the software Pilon (13) version 1.24, and assembly statistics were assessed using Benchmarking Universal Single-Copy Orthologs (14) version 4.0.2. Unless specified, default parameters were used for all the software.

*M. lentus* (*S. lentus*) strain B3 had a genome of 2,813,148 base pairs and a GC content of 32.0%. The genome was annotated by using the online RAST server (15), https://rast.nmpdr.org/, with manual curation, which identified 3,790 genes encompassing 3,711 coding DNA sequences, 22 rRNAs, and 57 tRNAs.

## Data Availability

The complete genome sequence of *M. lentus* (*S. lentus*) strain B3 was deposited in GenBank under the accession number CP180174. The associated BioProject and BioSample accession numbers are PRJNA796456 and SAMN24861188, respectively.
